# Stress‐induced changes in color expression mediated by iridophores in a polymorphic lizard

**DOI:** 10.1002/ece3.3349

**Published:** 2017-09-07

**Authors:** Anna C. Lewis, Katrina J. Rankin, Andrew J. Pask, Devi Stuart‐Fox

**Affiliations:** ^1^ School of BioSciences The University of Melbourne Parkville Vic Australia

**Keywords:** carotenoid, corticosterone, dermal chromatophore unit, pteridine, structural color

## Abstract

Stress is an important potential factor mediating a broad range of cellular pathways, including those involved in condition‐dependent (i.e., honest) color signal expression. However, the cellular mechanisms underlying the relationship between stress and color expression are largely unknown. We artificially elevated circulating corticosterone levels in male tawny dragon lizards, *Ctenophorus decresii*, to assess the effect of stress on the throat color signal. Corticosterone treatment increased luminance (paler throat coloration) and decreased the proportion of gray, thereby influencing the gray reticulations that produce unique patterning. The magnitude of change in luminance for corticosterone‐treated individuals in our study was around 6 “just noticeable differences” to the tawny dragon visual system, suggesting that lizards are likely to be able to perceive the measured variation. Transmission electron microscopy (TEM) of iridophore cells indicated that luminance increased with increasing density of iridophore cells and increased spacing (and/or reduced size) of crystalline guanine platelets within them. Crystal spacing within iridophores also differed between skin colors, being greater in cream than either gray or yellow skin and greater in orange than yellow skin. Our results demonstrate that stress detectably impacts signal expression (luminance and patterning), which may provide information on individual condition. This effect is likely to be mediated, at least in part, by structural coloration produced by iridophore cells.

## 1 INTRODUCTION

Colorful structures are often used as honest signals of individual quality (health, condition, performance; Kemp & Grether, 2014). Substantial research effort has been devoted to understanding the information on individual quality conveyed by color signals and how signal honesty is maintained. This research has focused disproportionately on carotenoid pigments because of their many physiological functions, particularly as antioxidants and in the immune response, and potential trade‐offs regarding carotenoid allocation to ornamentation (reviewed in Olson & Owens, 1998; Svensson & Wong, 2011). However, animal coloration is not produced by pigments alone, but by the interaction between the optical effects of pigments, which differentially absorb certain wavelengths of light, and structural mechanisms, whereby light is scattered and reflected by the nanometre‐scale structures in cells and tissues (Grether, Kolluru, & Nersissian, 2004). It is the interaction between pigmentary and structural components that results in the color manifested; therefore, understanding the mechanisms maintaining signal honesty depends critically on understanding costs and environmental effects on both components (Grether et al., 2004; Kemp, Herberstein, & Grether, 2011).

One of the most ubiquitous and important factors affecting individual condition, and potentially color expression, is stress. Physiological stress, both chronic and acute (e.g., due to food or water restriction, parasites, injury), triggers the secretion of glucocorticoid hormones such as corticosterone, the primary glucocorticoid in birds, reptiles, and many mammals (Wingfield et al., 1998). Corticosterone affects a wide range of essential physiological processes, including energy distribution and the immune response (Buchanan, 2000). Thus, stress may simultaneously affect coloration by altering the cellular processes involved in the deposition of pigments and intracellular structures, and an individual's response will be dependent on condition, defined as “the capacity to maintain vital cellular processes” (Hill, 2011). Consequently, color signals may be reliable indicators of an individual's condition and ability to tolerate stress. Evidence for this hypothesis derives from experiments showing that an increase in glucocorticoids can affect the size and intensity of sexually selected color signals (Fitze et al., 2009; Kemp & Rutowski, 2007; Kemp, Vukusic, & Rutowski, 2006; Lendvai, Bókony, Angelier, Chastel, & Sol, 2013; Roulin et al., 2008; Saino et al., 2013; San‐Jose & Fitze, 2013; San‐Jose, Granado‐Lorencio, Sinervo, & Fitze, 2013; Steffen & McGraw, 2009). However, whether stress affects processes of pigment production and/or deposition, the structural properties of cells and tissues, or both is largely unknown. The few studies that disentangle environmental effects, including stress, on pigments versus structural components of the same color patch have shown the latter to be partly or wholly responsible for environmentally induced variation (Jacot, Romero‐Diaz, Tschirren, Richner, & Fitze, 2010; Kemp & Rutowski, 2007; San‐Jose et al., 2013).

In poikilothermic vertebrates (fish, amphibians, and reptiles), coloration is produced by the arrangement and interaction of three primary pigment cell (chromatophore) types that contain either light‐absorbing pigments or light‐reflecting crystalline guanine platelets (Bagnara & Matsumoto, 2006; Bagnara, Taylor, & Hadley, 1968). The three chromatophore types typically appear in distinct layers, forming the dermal chromatophore unit (Bagnara et al., 1968). The three layers of chromatophores comprise: the xanthophores, containing red to yellow pigments such as carotenoids and pteridines; the iridophores, containing light‐reflecting and light‐scattering purine (mostly guanine) crystals; and a layer of dermal melanophores, containing eumelanin pigments that absorb the remaining light and affect the intensity of the color produced (Bagnara & Matsumoto 2006)(Figure 2006). Epidermal melanophores, similar in appearance and function although of a smaller size than dermal melanophores, also appear close to the epidermis (Bagnara & Matsumoto, 2006). Variation in the size, shape, orientation, and distribution of crystalline platelets within iridophores can produce a range of structural blue, green, gold, and red coloration, as well as altering the color produced by pigmentation (Kuriyama, Miyaji, Sugimoto, & Hasegawa, 2006; Morrison, 1995; Morrison, Rand, & Frost‐Mason, 1995; Morrison, Sherbrooke, & Frost‐Mason, 1996; Saenko, Teyssier, van der Marel, & Milinkovitch, 2013; Teyssier, Saenko, van der Marel, & Milinkovitch, 2015). For example, changes in the spacing of platelets rapidly produce color change in tree lizards (Morrison et al., 1996) and chameleons (Teyssier et al., 2015). Furthermore, in common lizards (*Lacerta vivipara*), corticosterone manipulation affects carotenoid‐based color expression by altering iridophore‐mediated “background reflectance,” but not carotenoid deposition in the skin (San‐Jose & Fitze, 2013).

**Figure 1:**
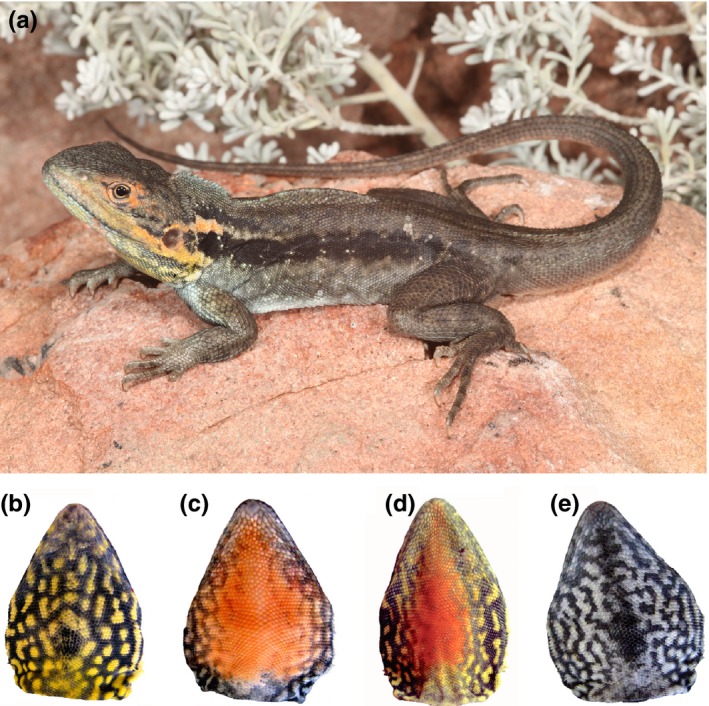
(a) The tawny dragon, *Ctenophorous decresii*, a small agamid lizard inhabiting rocky outcrops of South Australia. Photograph by Adam Elliott (b–e) The four throat color morphs of northern male *C. decresii*. ((b) **“**yellow” shows yellow coloration with gray patterning. (c) “orange” shows orange coloration with gray patterning. (d) **“**orange+yellow” shows a central orange patch surrounded by yellow coloration with gray patterning. (e) “gray” shows cream coloration with gray patterning)

The tawny dragon (*Ctenophorus decresii*) is a small agamid lizard inhabiting rocky outcrops of South Australia (Figure 1a). Males have conspicuously colored throats that they emphasize during male–male competition and courtship displays (Stuart‐Fox, Moussalli, Johnston, & Owens, 2004). Males in northern populations are polymorphic for throat color, with four discrete morphs: gray (dark gray and cream reticulation with no orange or yellow), yellow (varying amounts of yellow with cream and dark gray reticulations), orange (varying amounts of orange with cream and dark gray reticulations), and orange+yellow (orange center surrounded by yellow) (Figure 1b–e). The color morphs differ in behavior (Yewers, Pryke, & Stuart‐Fox, 2016) and associated endocrine levels (Yewers, Jessop, & Stuart‐Fox, 2017). Although color morphs and color expression (amount of orange or yellow) are heritable, there is nevertheless substantial variation between individuals in the extent of coloration and patterning within each morph category (Rankin, McLean, Kemp, & Stuart‐Fox, 2016). Such color variation may explain the ability of *C. decresii* males to distinguish between familiar and unfamiliar rivals, allowing them to avoid costly fights (Osborne, 2005). Thus, color conveys a range of information in this species, from behavioral strategy to individual identity; yet the extent to which color expression is condition‐dependent or influenced by stress remains unknown. In this study, we artificially elevated corticosterone levels and examined the effect on the chromatic and luminance properties. To identify cellular mechanisms, we quantified the effect of iridophore density and platelet spacing from transmission electron microscopy on perceived skin colors (orange, yellow, dark gray, and cream).

## 2 MATERIALS AND METHODS

### 2.1 Animal housing

Twenty‐three male *C. decresii* were used for this study, bred in captivity to parents captured in October and November 2011 from Warren Gorge, South Australia (31.4222° S, 138.7050° E). After hatching in November 2012 to January 2013, lizards were housed individually in the animal facility at the University of Melbourne in 55 L × 34 W × 38 D cm opaque plastic tubs. Each enclosure contained a layer of sand, a ceramic or terracotta tile hide, and a suspended heat lamp allowing animals to reach their preferred body temperatures (approx. 36°C; S. Walker, unpublished data). Room temperatures and lighting regimes were maintained at levels mimicking natural seasonal variation, with UV lights (05.10d Outback max 10.0 UV fluorescent tube; Ultimate Reptile Suppliers, South Australia) arranged above each enclosure (30 cm), emitting UVA and UVB radiation (10% at 30 cm). Lizards were misted and provided with live crickets three times per week. At sexual maturity, each lizard's throat color morph was visually identified as orange (*n* = 6), yellow (*n* = 9), orange + yellow (*n* = 3), or gray (*n* = 5) (Figure 1b–e).

### 2.2 Corticosterone implantation

Lizards were divided into two treatment groups, “cort” (*n* = 12) and “control” (*n* = 11). Those in the “cort” group had corticosterone levels artificially elevated using surgical implants, while “control” lizards were left untreated. Our goal was to ensure that the two groups differed in circulating corticosterone; therefore, “control” lizards were not sham‐implanted, to ensure that “control” lizards would not exhibit an increase in corticosterone levels due to the stress of minor surgery. We did not include a third sham‐implanted treatment group due to the limited number of individuals available for this study. Studies on other lizard species have confirmed that increased circulating corticosterone levels resulting from implants are due to the corticosterone powder rather than empty Silastic tubing implants (De Fraipont, Clobert, John‐Alder, & Meylan, 2000; DeNardo & Sinervo, 1994; French, McLemore, Vernon, Johnston, & Moore, 2007; Miles, Calsbeek, & Sinervo, 2007; Weiss, Mulligan, Wilson, & Kabelik, 2013). Nevertheless, it is possible that differences in color could be due to unmeasured effects of the minor surgical procedure rather than to corticosterone increases. However, this seems highly unlikely given that the small, subcutaneous incision caused no bleeding or inflammation and given the large differences in circulating corticosterone between treatment and control groups (see Section 2.1).

“Cort” lizards were first sterilized dorsally by wiping with 70% ethanol and injected subcutaneously with 0.2 ml of lidocaine (lignocaine hydrochloride 2%; Cenvet, Kings Park, NSW, Australia) and cooled on ice to induce immobility. A small dorsolateral incision was made near the shoulder through which sterile Silastic implants (5 mm lengths, 1.47 mm internal diameter, 1.96 mm external diameter; Dow Corning, Midland, MI, USA) packed with 2 mg of crystalline corticosterone powder (Sigma), and closed with 1‐mm Silastic adhesive at either end. The incision was closed with medical adhesive silicone (Dow Corning, Midland, MI, USA). After implantation, the animals were monitored until they showed normal breathing, righting and palpebral, toe‐pinch and tail‐pinch responses.

### 2.3 Corticosterone assays

Blood samples (50–100 μl) were taken from all animals at three time points (4 days prior to implantation, and 1 week and 4 weeks post‐implantation) by venipuncture from the vena angularis (in the corner of the mouth), within 1 min of capture. Plasma was separated from whole blood by centrifugation and frozen at −20°C until assayed. Plasma samples were assayed for corticosterone using an enzyme immunoassay kit (Cayman Chemical, Ann Arbor, MI, USA). Corticosterone assay plates, with standards and samples, were prepared in duplicate to manufacturer's instructions. Absorbance was measured at 405 nm on a plate reader (BioTek ELx800UV; BioTek Instruments, Winooski, VT, USA) using Gen5 v.2.05 software (BioTek Instruments, VT, USA). The intra‐ and interassay coefficients of variation were 1.85% and 18.11%, respectively, and sensitivity was 30 pg/ml.

### 2.4 Measuring color expression

Color expression of each lizard was measured over four weeks through weekly digital images of the throat taken under standardized light conditions and spectral reflectance measurements. Images were used to quantify pattern (the proportion of gray, orange, and yellow on the throat), while spectral measurements were used to quantify color. Images were taken using a Canon PowerShot SX1‐IS digital camera set to macro mode and calibrated with respect to radiance and light intensity (Stevens, Párraga, Cuthill, Partridge, & Troscianko, 2007). Camera responses (R, G, B, and luminance values) were linearized in relation to six gray‐scale squares of a colour checker standard (X‐Rite Inc., Grand Rapids, MI, USA) and their measured reflectance values (Garcia, Dyer, Greentree, Spring, & Wilksch, 2013). To remove the effect of minor variation in illumination, RGB values were equalized relative to the gray photographic standard nearest to the throat in each image (Stevens et al., 2007). Details of the linearization and equalization procedure are given in Ref. (Stevens et al. (2007), Garcia et al. (2013), and Smith et al. (2016)). Segmentation analysis was used to quantify the amount of gray, orange, and yellow coloration on the throat of each animal as described in Ref. (Teasdale, Stevens, and Stuart‐Fox (2013), and Rankin and Stuart‐Fox (2015)). This analysis identified regions of the throat as gray, orange, or yellow based on the RGB values of each pixel according to set threshold values of 0.20 for orange and 0.15 for yellow. A higher threshold was set for orange to ensure that orange and yellow throat coloration was clearly distinguished. Image calibration and segment analyses were performed in MATLAB (The MathWorks, Inc., MA, USA) using modified scripts written by John Endler and Martin Stevens.

Reflectance measurements were taken weekly using an Ocean Optics JAZ spectrometer and PX‐3 Pulsed Xenon light source connected to a probe via a bifurcated fiber‐optic cable. Measurements were of an oval point sample 3 × 4 mm, and taken relative to a 99% Spectralon diffuse white reflectance standard (Labsphere, NH, USA). Measurements were taken of multiple regions of the throat at a 45° angle in the range of 300–700 nm, the visual spectrum for most lizards (Loew, Fleishman, Foster, & Provencio, 2002; Olsson, Stuart‐Fox, & Ballen, 2013; Yewers et al., 2015). Each measured region was classified as being either dark gray, orange, yellow, or cream based on its spectral properties and appearance. We calculated chromatic (color) and achromatic (luminance) contrasts against a neutral background (white—uniform 99% reflectance) for each region. This enabled us to assess both the nature and degree of change. We applied the receptor noise limited (RNL) model (Vorobyev & Osorio, 1998; Vorobyev, Osorio, Bennett, Marshall, & Cuthill, 1998) to estimate the perceptual distance, measured in units of Just Noticeable Differences (JNDs), between skin color and the neutral white background, based on the likely visual system of *C. decresii*. We assumed an irradiance spectrum of full sunlight (Rottman, Floyd, & Viereck, 2004). Photoreceptor spectral sensitivities of UVS λ_max_ = 365 nm, SWS λ_max_ = 440 nm, MWS λ_max_ = 493 nm, LWS λ_max_ = 571 nm were based on information for the closely related ornate dragon, *Ctenophorus ornatus* (Barbour et al., 2002), as well as *C. decresii* visual systems, which includes a UVS cone (Yewers et al., 2015). We assumed that photoreceptor noise (ω_i_) for the LWS photoreceptor was 0.1 and derived the (ω_i_) of remaining photoreceptor classes using a ratio of 1:1:3.5:6 based on the relative photoreceptor frequencies in Barbour et al. (2002). Achromatic contrast was calculated based on the LWS photoreceptor, assuming ω_I_ = 0.05 and was log‐transformed for statistical analyses. Full details of visual modeling calculations are given in Teasdale et al. (2013) and McLean, Moussalli, and Stuart‐Fox (2014). Achromatic contrast values were log‐transformed to meet model assumptions.

### 2.5 Transmission electron microscopy sample preparation

Four weeks after treatment, the animals were humanely killed according to ethics guidelines using pentobarbitone. We randomly chose a subset of 13 individuals from which we dissected 24 tissue samples (3 mm^2^) representing the different skin colors (seven orange, eight yellow, four dark gray, and five cream). As pigment cell densities, and especially iridophores, are likely to differ between skin colors, we focused on skin color rather than morph, treating the same skin color from different morphs as equivalent (there is no difference in the spectral properties of orange or yellow between morphs). Nevertheless, we ensured that morphs were approximately equally distributed between treatment groups.

Samples were fixed in 2.5% glutaraldehyde in phosphate‐buffered saline (PBS) for 4 hr at room temperature. Fixed tissue samples were rinsed three times in PBS for 10 min each, before being dehydrated in increasing concentrations of ethanol consisting of 10%, 30%, 50%, 70%, 90%, 100%, and 100% anhydrous ethanol for 60 min each. Following dehydration, the cells were infiltrated with increasing concentrations of LR White resin in ethanol consisting of 25%, 50%, 75%, and 100% resin for 6 hr each step. After a second change of 100% resin, the samples were embedded in fresh resin in gelatine capsules and allowed to gently sink to the bottom. The gelatine capsules were capped to exclude air and the resin polymerized in an oven at 60°C for 24 h.

The embedded tissues in resin blocks were sectioned with a diamond knife on a Leica Ultracut S microtome and ultrathin sections (90 nm) were collected onto formvar‐coated 100 mesh hexagonal copper grids. The sections on grids were sequentially stained with saturated uranyl acetate for 10 min and Triple Lead Stain for 5 min (Sato, 1968) and viewed in an FEI Tecnai Spirit transmission electron microscope at 120 kV. Images were captured with a Gatan Eagle digital camera, at a resolution of 2048 × 2048 pixels.

### 2.6 Iridophore density

**Figure 2:**
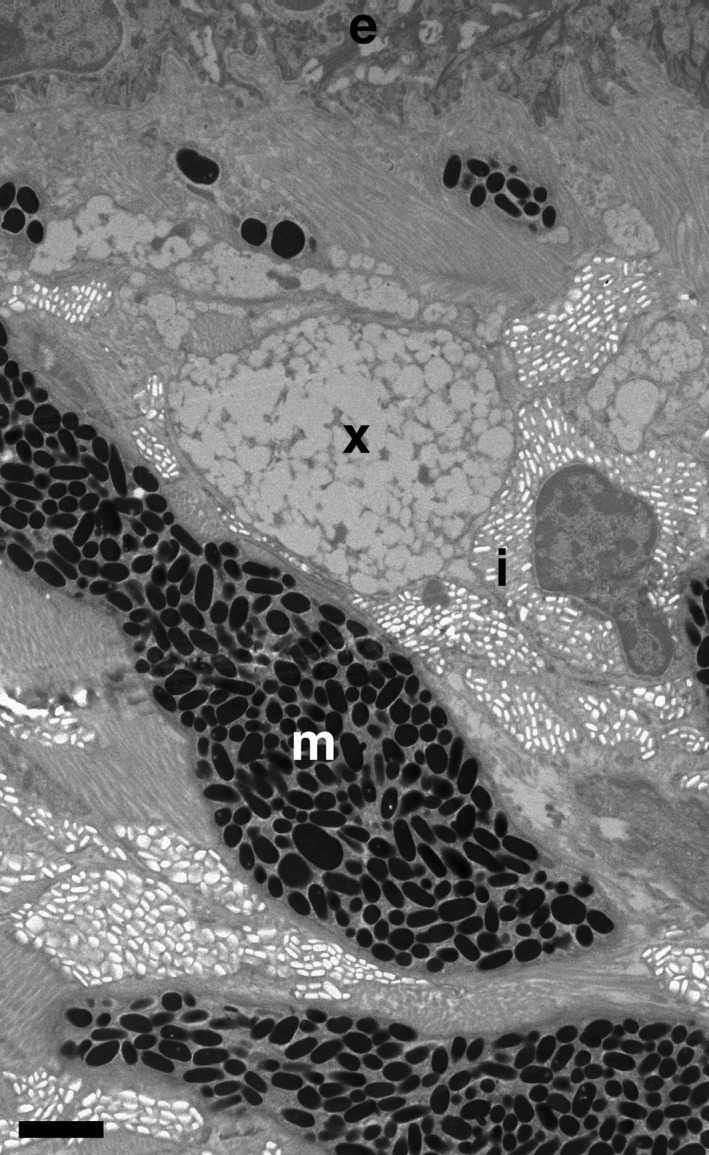
TEM micrograph of the spatial arrangement of chromatophores in the dermal chromatophore unit. The epidermis (e), iridophores (i), melanophores (m), and xanthophore layer containing xanthophores and collagen bundles (x) are indicated. Bar = 2 μm

Images were taken at low magnification (1650×) to measure the density of iridophores in different skin colors up to 15 μm from the epidermis. At this magnification, iridophores and melanophores could be easily recognized but xanthophores could not be reliably distinguished from bundles of collagen (Figures 2, S1). Furthermore, melanophores were relatively rare. We measured the length of intersections of iridophores with a series of 15‐μm vertical transects (*n* = 539, 15–36 transects per skin sample), spaced 5 μm apart within each analyzable section of the sample. To determine iridophore density, we measured the proportion of each transect that intersected with iridophores, deriving a median proportion across the 15–36 transects, depending on the size of the sample, for statistical analysis.

### 2.7 Iridophore platelet spacing

High magnification (6500× and 11,000×) images were taken to measure platelet spacing within iridophore cells. In some lizard species (e.g., spiny lizards, Morrison et al., 1995; whiptail lizards, Mathger, Land, Siebeck, & Marshall, 2003; plestiodons, Kuriyama et al., 2006; geckoes, Saenko et al., 2013; and chameleons, Teyssier et al., 2015), iridophore cells show a traditional lattice structure, where near‐identical platelets are evenly spaced throughout the cell. This is not the case in tawny dragon skin tissue, where there is too much variation to meaningfully characterize platelet arrangement based on measurements of platelet size and the intervening spaces. Instead, we measured the area of a series of 1 × 1 μm plots (*n* = 600, 21–28 per sample) taken up by platelets compared to cytoplasm (Figure S2). As the size of intact platelets and the holes left behind by platelets wholly or partially damaged by sample preparation were comparable, both were classified as iridophore platelets. Using ImageJ, the identified iridophore platelets were outlined in white and, following threshold adjustment, selected and measured using the wand tool (Figure S2). We used the median area of iridophore platelets per sample (from the 21–28 1 × 1 μm plots per sample) for statistical analyses. This is equivalent to % of the two‐dimensional area occupied by platelets relative to cytoplasm.

### 2.8 Statistical analysis

We analyzed the effect of implants on circulating corticosterone using a general linear mixed model (GLMM). Time (pre‐implantation, 1 week post‐implantation, and 4 weeks post‐implantation), treatment (cort, control), morph (orange, orange + yellow, yellow, or gray), and their interactions were included as fixed factors. The three‐way interaction term was removed from the final model if not significant. Lizard ID was included as a random factor to account for repeated measures on individuals.

To measure changes in throat coloration in response to corticosterone treatment, we analyzed the proportion of gray, orange, and yellow using general linear mixed models (GLMM), with time, treatment, and their interaction as fixed factors. The achromatic and chromatic contrast of specified points on the throat were analyzed using GLMMs with sample color, time, and treatment, as well as the interaction between treatment and time, and color and treatment as fixed factors. In all models, ID was included as a random factor to account for repeated measures over time.

To assess the difference between skin colors (orange, yellow, dark gray, and cream) in the median proportion of iridophores intersected by 15‐μm transects perpendicular to the epidermis, we used a GLMM with sample color as a fixed factor and ID as a random factor to account for 11 instances where more than one sample was taken from one individual. We also assessed the effect that the median proportions of iridophores within a sample have on sample achromatic and chromatic contrasts using GLMMs with individual ID as a random factor.

To analyze the spacing of iridophore platelets, which may alter to produce changes in color (Morrison et al., 1996; Teyssier et al., 2015), we used a GLMM with the median area of iridophore platelets as a dependent variable, sample color as a fixed factor and lizard ID as a random factor. The effect that iridophore platelet area had on achromatic and chromatic contrast was analyzed using a GLMM with achromatic or chromatic contrast as the dependent variable, sample color, platelet area, and their interaction as fixed factor and ID as a random factor.

All analyses were performed in SAS 9.4 (SAS Institute Inc.; PROC MIXED). Residual plots for all models were checked for normality to ensure model assumptions were met and effects were considered significant at *p* < .05.

## 3 RESULTS

### 3.1 Corticosterone assays

**Figure 3:**
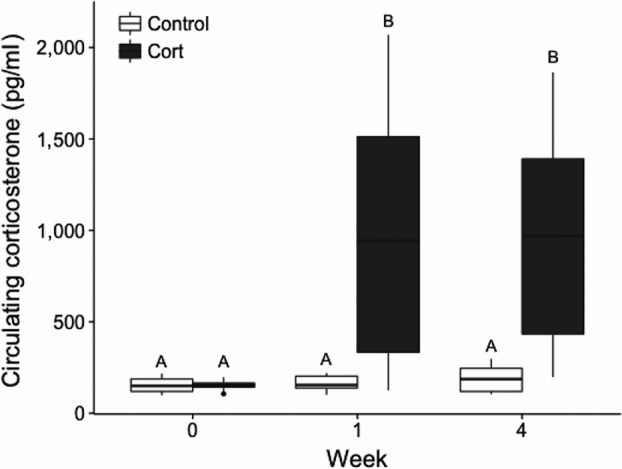
Effect of artificial elevation of corticosterone using Silastic implants. Mean circulating corticosterone levels in male *Ctenophorous decresii* pre‐implantation, and 1 week and 4 weeks post‐implantation. Different letters (A and B) indicate statistically different values.

Circulating corticosterone levels significantly increased over time in corticosterone‐treated individuals (*F*_2,42_ = 15.64, *p* < .0001). Corticosterone concentration in blood plasma increased from 153.5 ± 7.77 pg/ml pre‐implantation to 980.6 ± 200.74 pg/ml at one week post‐implantation (*t*_42_ = −7.21, *p* < .0001) and did not change further at 4 weeks post‐implantation (pre‐implantation vs. week 4: *t*_42_ = −7.20, *p* < .0001, week 1 vs. week 4: *t*_42_ = 0.01, *p* = 1), (Figure 3). Control individuals did not change in corticosterone concentration over the four weeks monitoring period (pre‐implantation vs. week 1: *t*_42_ = −0.08, *p* = 1, week 1 vs. week 4: *t*_42_ = −0.23, *p* = 1). Corticosterone levels did not significantly differ between color morphs (*F*_3,15_ = 14.84, *p* = .86).

### 3.2 Effect of corticosterone on skin color

Segmentation analysis of weekly photographs showed an effect of corticosterone treatment on the proportion of throat colors (Tables 1 and 2). There was a significant treatment by time interaction for the proportion of gray and orange throat coloration (Table 2). The proportion of gray throat coloration decreased significantly in corticosterone‐treated but not control individuals (Table 1). This was because the proportion of both yellow and orange in corticosterone‐treated individuals increased, while the proportion of orange in control individuals decreased (Table 1). However, the change in the proportion of yellow or orange coloration individually for either treatment group was not significant (Table 2).

**Table 1:** Percentages of gray, orange, and yellow throat coloration, and achromatic and chromatic contrast values at three time points. Values are least‐squared means ± SE. Control and cort values were not significantly different for any component of color at any one time point; however, gray coloration significantly decreased over time in cort‐treated individuals and not control individuals. The proportions of gray, orange, and yellow throat colors were derived from segmentation analysis of digital images. Chromatic contrast and achromatic contrast values were derived from spectral measurements

Dependent	Pretreatment	Week 1	Week 4
Gray (%)			
Cort	77.16 ± 6.92	73.31 ± 7.29	71.57 ± 7.68
Control	81.10 ± 5.03	78.84 ± 5.28	82.15 ± 4.50
Orange (%)			
Cort	14.94 ± 6.48	15.92 ± 6.68	16.19 ± 7.27
Control	9.94 ± 5.14	10.47 ± 5.41	7.98 ± 4.37
Yellow (%)			
Cort	7.90 ± 2.84	10.76 ± 3.93	12.24 ± 4.05
Control	8.94 ± 3.23	10.70 ± 3.60	9.88 ± 3.38
Chromatic contrast			
Cort	4.749 ± 0.32	4.022 ± 0.29	4.823 ± 0.31
Control	4.420 ± 0.33	4.161 ± 0.30	4.729 ± 0.31
Achromatic contrast			
Cort	19.518 ± 1.71	15.014 ± 1.51	13.253 ± 1.63
Control	15.651 ± 1.74	14.985 ± 1.57	15.018 ± 1.66

**Table 2:** Effect of corticosterone implantation on components of throat coloration. Gray, orange, and yellow indicate the proportion of those colors on the throat and were derived from segmentation analysis of digital images. Chromatic contrast and achromatic contrast values were derived from spectral measurements

Dependent	Fixed factor	*F*_df_	*p*‐value
Gray	Treatment	0.56_1,41_	.46
	Time	4.58_2,41_	.02
	Treatment × Time	5.61_2,41_	.007
Orange	Treatment	0.53_1,41_	.47
	Time	1.58_2,41_	.22
	Treatment × Time	3.65_2,41_	.035
Yellow	Treatment	0.01_1,41_	.93
	Time	4.46_2,41_	.02
	Treatment × Time	1.66_2,41_	.20
Chromatic contrast	Treatment	0.06_1,298_	.81
	Time	10.9_2,298_	<.0001
	Color	82.87_3,298_	<.0001
	Treatment × Time	1.04_2,298_	.35
	Treatment × Color	1.27_3,298_	.29
Achromatic contrast	Treatment	0.05_1,298_	.82
	Time	9.47_2,298_	.0001
	Color	73.64_3,298_	<.0001
	Treatment × Time	4.59_2,298_	.004
	Treatment × Color	4.65_3,298_	.01

Chromatic contrast decreased at week 1 then increased to pre‐implantation levels at week 4 for both treatment groups (Table 1, pre‐implantation vs. week 1: *t*_298_ = 3.01, *p* = .003; week 1 vs. week 4: t_298_ = −4.46, *p* < .0001; pre‐implantation vs. week 4: *t*_298_ = −1.09, *p* = .28). However, for achromatic contrast, there was a significant interaction between time and treatment (Table 2). Achromatic contrast (to a white background) significantly decreased in corticosterone‐treated individuals, indicating that throat color became paler by week 1, then remained stable at 4 weeks (Table 1, pre‐implantation vs. week 1: *t*_298_ = 3.92, *p* = .0001; pre‐implantation vs. week 4: *t*_298_ = 5.10, *p* < .0001; week 1 vs. week 4: *t*_298_ = 1.70, *p* = .091). The magnitude of change in achromatic contrast for corticosterone‐treated individuals in our study was around 6 JNDs, therefore clearly distinguishable by lizards. By contrast, there was no consistent change in achromatic contrast between the three time points in control individuals (Table 1). Despite the difference between corticosterone and control individuals in the change in achromatic contrast over time, at week 4, there was no significant difference between treatment groups in either chromatic contrast (cort vs. control: *t*_298_ = −0.21, *p* = .83) or achromatic contrast (cort vs. control: *t*_298_ = 1.46, *p* = .14). Consequently, there was no significant difference between treatment groups in the subset of samples selected for transmission electron microscopy (Table S1).

### 3.3 Relationship between iridophore density and skin color

**Figure 4:**
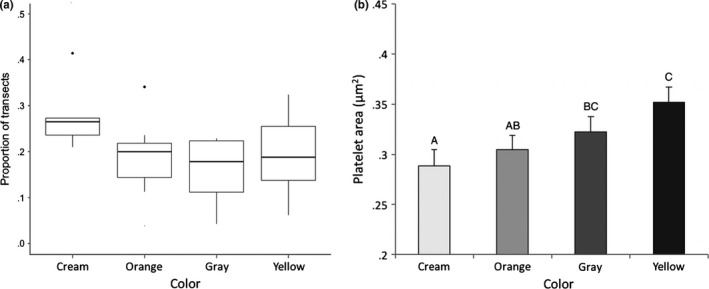
(a) The effect of sample color on the density of iridophores, based on the proportion of a 15‐μm transect (*N* = 539) that intersects with iridophore cells (proportion of transects). (b) Differences in the area of 1 × 1 μm plots taken up by platelets in iridophore cells between sample colors. Data are least‐squared mean ± SE. Different letters (a, b, and c) indicate statistically different values

**Figure 5:**
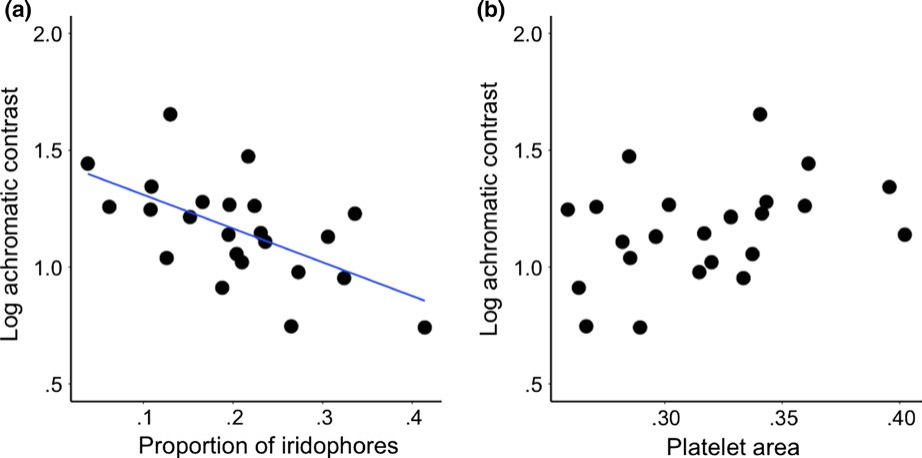
(a) Correlation between median proportion of iridophores per transect and achromatic contrast against a white background (i.e., higher achromatic contrast = lower luminance, lower achromatic contrast = higher luminance) (*R* ^2^ = 0.36). (b) Correlation between iridophore platelet spacing (the median area of platelets within 1 × 1 μm plots) and achromatic contrast (*R* ^2^ = 0.14, NS)

Iridophores were the most prevalent chromatophore from 5.6 to 15 μm from the epidermis, peaking at 7.2 μm where they intersected with 30.6% of transects. There were no significant overall differences between skin colors in the median proportions of iridophores that intersected with the 15‐μm transects (*F*_3,7_ = 1.96, *p* = .21; Figure 4a). However, there was a significant positive relationship between the median proportion of iridophores intersected by the 15‐μm transects and achromatic contrast (*F*_1,9_ = 11.64, *p* = .008; Figure 5a), but not chromatic contrast (*F*_1,9_ = 1.70, *p* = .22).

### 3.4 Relationship between iridophore spacing and skin color

Iridophore platelet area within 1 × 1 μm plots differed significantly between skin sample colors (*F*_3,584_ = 11.9, *p* < .0001) (Fig. S3). Platelets in cream samples took up 28.8 ± 1.62% of 1 × 1 μm plots, a significantly smaller percentage than in dark gray or yellow samples (cream vs. dark gray: *t*_583_ = −4.47, *p* < .0001, cream vs. yellow: *t*_583_ = −3.57, *p* < .0004) (Figure 4b), indicating that the platelets in cream skin were either smaller on average and/or more widely spaced. Iridophore cells in orange samples had a significantly lower area taken up by platelets than in yellow samples (orange vs. yellow: *t*_583_ = −3.78, *p* = .0002).

There was a weak trend for a relationship between achromatic contrast and median iridophore platelet area (*F*_1,9_ = 3.37, *p* = .1). Increasing contrast (i.e., decreasing luminance) was associated with an increase in platelet area (Figure 5b). Platelet area did not affect the chromatic contrast of skin samples (*F*_1,9_ = 0.61, *p* = .46).

## 4 DISCUSSION

Our results demonstrate that stress alters ornamental color expression in male tawny dragons. Throat color became paler (i.e., higher luminance), and the proportion of gray decreased in corticosterone‐treated but not control individuals. Analyses of TEM images indicated that luminance is associated primarily with the density of iridophore cells, but also the spacing of crystal platelets within them. As expected, luminance increased with an increasing median proportion of iridophores and a decrease in the area taken up by crystals, most likely caused by an increase in spacing between crystals and/or a decrease in their size. The area taken up by crystals within iridophores also differed between skin colors, being greater in cream than either dark gray or yellow skin and greater in orange than yellow skin. We cannot directly associate corticosterone‐induced changes in skin color with iridophore variation because there was no difference in the color (chromatic or achromatic contrast) of corticosterone‐treated and control individuals in the subset of samples randomly chosen for TEM analyses. However, our results suggest that effects of stress on ornamental coloration in this species are likely to be mediated, at least in part, by effects on the structural color produced by iridophores.

The increase in overall throat luminance following stress manipulation is consistent with results found in birds (Almasi, Roulin, Jenni‐Eiermann, & Jenni, 2008; Roulin et al., 2008), butterflies (Kemp & Rutowski, 2007), and other lizard species (San‐Jose & Fitze, 2013). The magnitude of change in achromatic contrast for corticosterone‐treated individuals in our study was around 6 JNDs, suggesting that lizards are likely to be able to perceive the observed variation. Furthermore, corticosterone had greatest impact on the proportion of dark gray reticulations that produce the unique patterning on tawny dragon throats. General lightening of throat coloration may also reduce the apparent contrast between different throat colors, causing gray patterning to appear less prominent. Therefore, the combined change in color and pattern that we observed in response to stress could be sufficient to convey information to mates and rivals on individual condition. Similarly, stress has been shown to alter both the size and luminance of dark coloration in birds (Almasi et al., 2008; Roulin et al., 2008) and other lizard species (San‐Jose & Fitze, 2013).

Changes in the density of iridophores and/or the spacing of crystalline platelets within them may mediate the stress‐induced change in luminance that we observed. Paler samples (higher luminance) intersected a higher proportion of iridophore cells, in which platelets were more widely spaced and/or smaller. Changes in the spacing of platelets can occur rapidly in response to increased osmotic pressure in chameleons (Teyssier et al., 2015) and temperature in tree lizards (Morrison et al., 1996), causing coloration to become lighter or bluer, respectively. However, unlike chameleons or tree lizards, platelets in tawny dragon skin tissue were highly irregular in size, shape, and orientation (although quasi‐ordered), which would produce incoherent scattering of light across all visible wavelengths (Vukusic, Hallam, & Noyes, 2007). Our data suggest that one or both mechanisms (change in iridophore density and change in crystal platelet spacing) may be involved in the change in luminance. The relative importance of these mechanisms may depend on time scale; for example, change in platelet spacing could occur relatively rapidly in response to acute stress, while changes in pigment cell density (or pigment synthesis/deposition) in response to chronic stress may occur over a longer time scale of days, weeks, or months.

Elevation of corticosterone levels appeared to have little effect on the intensity or proportion of orange or yellow coloration. Effects of stress on red to yellow coloration may depend on pigments used to generate these colors. The two classes of pigments used to generate red to yellow coloration in poikilothermic vertebrates are carotenoids and pteridines (Bagnara & Matsumoto, 2006). Both classes of pigment are found in all skin colors in tawny dragon lizards (McLean, Lutz, Rankin, Stuart‐Fox, & Moussalli, 2017). Animals acquire carotenoids exclusively from the diet; whereas colored pteridines within pigment cells are synthesized de novo from purines, which are presumed to be nonlimiting (Braasch, Schartl, & Volff, 2007; Svensson & Wong, 2011; Ziegler, 2003). Stress limits the size and color of pteridine‐based orange ornamentation in striped plateau lizards (Weiss, Foerster, & Hudon, 2012; Weiss et al., 2013). Carotenoid‐based coloration, however, is highly variable in its response to corticosterone which may have no effect (Fitze et al., 2009; in lizards), a negative effect (Loiseau, Fellous, Haussy, Chastel, & Sorci, 2008; in birds) or a positive effect (McGraw, Lee, & Lewin, 2011; Kennedy, Lattin, Romero, & Dearborn, 2013; in birds) although this may vary taxonomically. This may be because the relationship between stress and carotenoid‐based color expression is influenced by the competing physiological functions of carotenoids and consequent trade‐offs in carotenoid allocation (Fairhurst, Dawson, van Oort, & Bortolotti, 2014). When orange or yellow coloration is produced by both carotenoid and pteridine pigments, as is the case in tawny dragons (McLean et al., 2017), it is possible that their relative contribution could vary, depending on stress, although this idea has not been investigated to our knowledge. Furthermore, the corticosterone dose used in our implants was relatively low compared to those used by Weiss et al. (2013), and may have been insufficient to cause changes in either pteridine synthesis or carotenoid deposition.

Levels of circulating corticosterone post‐implantation were also much lower than those found in wild tawny dragon populations (Jessop et al., 2016). Despite these elevated stress levels, tawny dragons in the wild are able to produce conspicuous coloration, which indicates that there may be other environmental effects on color expression and/or significant differences in the ability of captive and wild animals to cope with stress. Alternatively, captive tawny dragons that have reduced breeding opportunities may more readily allocate resources away from producing ornamental coloration. *Ctenophorus decresii* is polymorphic for male throat coloration, but color morphs do not differ in their response to capture stress (Yewers et al., 2017) despite their differences in aggressiveness and boldness (Yewers et al., 2016). Consistent with this, color morphs did not differ in effects of stress on color expression, with effects observed across skin colors within and between morphs. Thus, stress may be a general mechanism influencing signal honesty in this species, irrespective of morph‐specific behavioral strategies.

The study of the cellular mechanisms underlying color expression is important to understand both the information conveyed by signals and mechanisms maintaining their honesty. Stress may be a particularly important mechanism mediating condition‐dependent (i.e., honest) color expression, particularly structural coloration (Kemp et al., 2006; McGraw, Mackillop, Dale, & Hauber, 2002; San‐Jose et al., 2013). Our results demonstrate that stress impacts ornamental coloration in a detectable way that may provide information on individual condition by influencing overall luminance as well as patterning. This effect is likely to be mediated, at least in part, by changes in iridophore cells producing structural coloration. Disentangling the effects of stress on the complex systems of color production involves an integrative approach investigating pigment cell structures, the physiological processes that affect them, and the ultimate ecological and evolutionary consequences. Ideally, this requires examination of both pigmentary and structural components and their interaction over time, and how these are altered in response to external and internal factors. The dermal chromatophore unit in poikilothermic vertebrates provides a promising model in this regard.

## DATA ACCESSIBILITY

Data will be made available on the Dryad Repository upon acceptance.

## CONFLICT OF INTEREST

None declared.

## AUTHOR CONTRIBUTIONS

DSF, KJR and AJP conceived the ideas; all authors designed methodology; ACL collected the data; ACL and DSF analyzed the data; ACL and DSF led the writing of the manuscript. All authors contributed critically to the drafts and gave final approval for publication.

## References

[ece33349-bib-0001] Almasi, B. , Roulin, A. , Jenni‐Eiermann, S. , & Jenni, L. (2008). Parental investment and its sensitivity to corticosterone is linked to melanin‐based coloration in barn owls. Hormones and Behavior, 54, 217–223. 18413149 10.1016/j.yhbeh.2008.02.021

[ece33349-bib-0002] Bagnara, J. T. , & Matsumoto, J. (2006). Comparative anatomy and physiology of pigment cells in nonmammalian tissues In The Pigmentary System: Physiology and Pathophysiology, Second Edition Nordlund J. J., Boissy R. E., Hearing V. J., King R. A., Oetting W. S. and Ortonne J.‐P. (Eds.), (pp. 11–59). Oxford, UK: Blackwell Publishing Ltd.

[ece33349-bib-0003] Bagnara, J. T. , Taylor, J. D. , & Hadley, M. E. (1968). The dermal chromatophore unit. Journal of Cell Biology, 38, 67–79. 5691979 10.1083/jcb.38.1.67 PMC2107474

[ece33349-bib-0004] Barbour, H. R. , Archer, M. A. , Hart, N. S. , Thomas, N. , Dunlop, S. A. , Beazley, L. D. , & Shand, J. (2002). Retinal characteristics of the ornate dragon lizard, *Ctenophorus ornatus* . Journal of Comparative Neurology, 450, 334–344. 12209847 10.1002/cne.10308

[ece33349-bib-0005] Braasch, I. , Schartl, M. , & Volff, J.‐N. (2007). Evolution of pigment synthesis pathways by gene and genome duplication in fish. BMC Evolutionary Biology, 7, 74. 17498288 10.1186/1471-2148-7-74 PMC1890551

[ece33349-bib-0006] Buchanan, K. L. (2000). Stress and the evolution of condition‐dependent signals. Trends in Ecology and Evolution, 15, 156–160. 10717685 10.1016/s0169-5347(99)01812-1

[ece33349-bib-0007] De Fraipont, M. , Clobert, J. , John‐Alder, H. , & Meylan, S. (2000). Increased pre‐natal maternal corticosterone promotes philopatry of offspring in common lizards *Lacerta vivipara* . Journal of Animal Ecology, 69, 404–413.

[ece33349-bib-0008] DeNardo, D. F. , & Sinervo, B. (1994). Effects of corticosterone on activity and home‐range size of free‐ranging male lizards. Hormones and Behavior, 28, 53–65. 8034282 10.1006/hbeh.1994.1005

[ece33349-bib-0009] Fairhurst, G. D. , Dawson, R. D. , van Oort, H. , & Bortolotti, G. R. (2014). Synchronizing feather‐based measures of corticosterone and carotenoid‐dependent signals: What relationships do we expect? Oecologia, 174, 689–698. 24233689 10.1007/s00442-013-2830-5

[ece33349-bib-0010] Fitze, P.S. , Cote, J. , San‐Jose, L. M. , Meylan, S. , Isaksson, C. , Andersson, S. , … Clobert, J. (2009). Carotenoid‐based colours reflect the stress response in the common lizard. PLoS ONE, 4(4), e5111. 19352507 10.1371/journal.pone.0005111 PMC2663031

[ece33349-bib-0011] French, S. S. , McLemore, R. , Vernon, B. , Johnston, G. I. H. , & Moore, M. C. (2007). Corticosterone modulation of reproductive and immune systems trade‐offs in female tree lizards: Long‐term corticosterone manipulations via injectable gelling material. Journal of Experimental Biology, 210, 2859–2865. 17690234 10.1242/jeb.005348

[ece33349-bib-0012] Garcia, J. E. , Dyer, A. G. , Greentree, A. D. , Spring, G. , & Wilksch, P. A. (2013). Linearisation of RGB camera responses for quantitative image analysis of visible and UV photography: A comparison of two techniques. PLoS ONE, 8(11), e79534. 24260244 10.1371/journal.pone.0079534 PMC3832603

[ece33349-bib-0013] Grether, G. F. , Kolluru, G. R. , & Nersissian, K. (2004). Individual colour patches as multicomponent signals. Biological Reviews, 79, 583–610. 15366764 10.1017/s1464793103006390

[ece33349-bib-0014] Hill, G. E. (2011). Condition‐dependent traits as signals of the functionality of vital cellular processes. Ecology Letters, 14, 625–634. 21518211 10.1111/j.1461-0248.2011.01622.x

[ece33349-bib-0015] Jacot, A. , Romero‐Diaz, C. , Tschirren, B. , Richner, H. , & Fitze, P. S. (2010). Dissecting carotenoid from structural components of carotenoid‐based coloration: a field experiment with great tits (*Parus major*). American Naturalist, 176, 55–62. 10.1086/653000 20470031

[ece33349-bib-0016] Jessop, T. S. , Lane, M. L. , Teasdale, L. , Stuart‐Fox, D. , Wilson, R. S. , Careau, V. , & Moore, I. T. (2016). Multiscale evaluation of thermal dependence in the glucocorticoid response of vertebrates. American Naturalist, 188, 342–356. 10.1086/687588 27501091

[ece33349-bib-0017] Kemp, D. J. , & Grether, G. F. (2014) Integrating functional and evolutionary approaches to the study of color‐based animal signals In Animal Signaling and Function: An Integrative Approach Irschick D. J., Briffa M. & Podos J. (Eds), (pp. 111–140). Hoboken, NJ, USA: John Wiley & Sons, Inc.

[ece33349-bib-0018] Kemp, D. J. , Herberstein, M. E. , & Grether, G. F. (2011). Unraveling the true complexity of costly color signaling. Behavioral Ecology, 23, 233–236.

[ece33349-bib-0019] Kemp, D. J. , & Rutowski, R. L. (2007). Condition dependence, quantitative genetics, and the potential signal content of iridescent ultraviolet butterfly coloration. Evolution, 61, 168–183. 17300436 10.1111/j.1558-5646.2007.00014.x

[ece33349-bib-0020] Kemp, D. J. , Vukusic, P. , & Rutowski, R. L. (2006). Stress‐mediated covariance between nano‐structural architecture and ultraviolet butterfly coloration. Functional Ecology, 20, 282–289.

[ece33349-bib-0021] Kennedy, E. A. , Lattin, C. R. , Romero, L. M. , & Dearborn, D. C. (2013). Feather coloration in museum specimens is related to feather corticosterone. Behavioral Ecology and Sociobiology, 67, 341–348.

[ece33349-bib-0022] Kuriyama, T. , Miyaji, K. , Sugimoto, M. , & Hasegawa, M. (2006). Ultrastructure of the dermal chromatophores in a lizard (Scincidae: *Plestiodon latiscutatus*) with conspicuous body and tail coloration. Zoological Science, 23, 793–799. 17043401 10.2108/zsj.23.793

[ece33349-bib-0023] Lendvai, Á. Z. , Bókony, V. , Angelier, F. , Chastel, O. , & Sol, D. (2013). Do smart birds stress less? An interspecific relationship between brain size and corticosterone levels. Proceedings Biological Sciences/The Royal Society, 280, 1–8. 10.1098/rspb.2013.1734 PMC3779331 24026820

[ece33349-bib-0024] Loew, E. R. , Fleishman, L. J. , Foster, R. G. , & Provencio, I. (2002). Visual pigments and oil droplets in diurnal lizards: A comparative study of Caribbean anoles. Journal of Experimental Biology, 205, 927–938. 11916989 10.1242/jeb.205.7.927

[ece33349-bib-0025] Loiseau, C. , Fellous, S. , Haussy, C. , Chastel, O. , & Sorci, G. (2008). Condition‐dependent effects of corticosterone on a carotenoid‐based begging signal in house sparrows. Hormones and Behavior, 53, 266–273. 18029282 10.1016/j.yhbeh.2007.10.006

[ece33349-bib-0026] Mathger, L. M. , Land, M. , Siebeck, U. , & Marshall, N. (2003). Rapid colour changes in multilayer reflecting stripes in the paradise whiptail, *Pentapodus paradiseus* . Journal of Experimental Biology, 206, 3607–3613. 12966052 10.1242/jeb.00599

[ece33349-bib-0027] McGraw, K. J. , Lee, K. , & Lewin, A. (2011). The effect of capture‐and‐handling stress on carotenoid‐based beak coloration in zebra finches. Journal of Comparative Physiology A: Neuroethology, Sensory, Neural, and Behavioral Physiology, 197, 683–691. 10.1007/s00359-011-0631-z 21344204

[ece33349-bib-0028] McGraw, K. J. , Mackillop, E. A. , Dale, J. , & Hauber, M. E. (2002). Different colors reveal different information: How nutritional stress affects the expression of melanin‐ and structurally based ornamental plumage. Journal of Experimental Biology, 205, 3747–3755. 12409501 10.1242/jeb.205.23.3747

[ece33349-bib-0029] McLean, C. A. , Lutz, A. , Rankin, K. J. , Stuart‐Fox, D. , & Moussalli, A. (2017). Revealing the biochemical and genetic basis of colour variation in a polymorphic lizard. Molecular Biology and Evolution, online in advance of print. 10.1093/molbev/msx136 28431132

[ece33349-bib-0030] McLean, C. A. , Moussalli, A. , & Stuart‐Fox, D. (2014). Local adaptation and divergence in colour signal conspicuousness between monomorphic and polymorphic lineages in a lizard. Journal of Evolutionary Biology, 27, 2654–2664. 25330209 10.1111/jeb.12521

[ece33349-bib-0031] Miles, D. B. , Calsbeek, R. , & Sinervo, B. (2007). Corticosterone, locomotor performance, and metabolism in side‐blotched lizards (*Uta stansburiana*). Hormones and Behavior, 51, 548–554. 17379216 10.1016/j.yhbeh.2007.02.005

[ece33349-bib-0032] Morrison, R. L. (1995). A transmission electron microscopic (TEM) method for determining structural colors reflected by lizard iridophores. Pigment Cell Research, 8, 28–36. 7792252 10.1111/j.1600-0749.1995.tb00771.x

[ece33349-bib-0033] Morrison, R. L. , Rand, M. S. , & Frost‐Mason, S. K. (1995). Cellular basis of color differences in three morphs of the lizard *Sceloporus undulatus* erythrocheilus. Copeia, 1995, 397–408.

[ece33349-bib-0034] Morrison, R. L. , Sherbrooke, W. C. , & Frost‐Mason, S. K. (1996). Temperature‐sensitive, physiologically active iridophores in the lizard *Urosaurus ornatus*: An ultrastructural analysis of color change. Copeia, 1996, 804.

[ece33349-bib-0035] Olson, V. A. , & Owens, I. P. F. (1998). Costly sexual signals: Are carotenoids rare, risky or required? Trends in Ecology and Evolution, 13, 510–514. 21238418 10.1016/s0169-5347(98)01484-0

[ece33349-bib-0036] Olsson, M. , Stuart‐Fox, D. , & Ballen, C. (2013). Genetics and evolution of colour patterns in reptiles. Seminars in Cell & Developmental Biology, 24, 529–541. 23578866 10.1016/j.semcdb.2013.04.001

[ece33349-bib-0037] Osborne, L. (2005). Rival recognition in the territorial tawny dragon (*Ctenophorus decresii*). Acta Ethologica, 8, 45–50.

[ece33349-bib-0038] Rankin, K. J. , McLean, C. A. , Kemp, D. J. , & Stuart‐Fox, D. (2016). The genetic basis of discrete and quantitative colour variation in the polymorphic lizard, *Ctenophorus decresii* . BMC Evolutionary Biology, 16, 179. 27600682 10.1186/s12862-016-0757-2 PMC5012029

[ece33349-bib-0039] Rankin, K. , & Stuart‐Fox, D. (2015). Testosterone‐induced expression of male colour morphs in females of the polymorphic tawny dragon lizard, *Ctenophorus decresii* . PLoS ONE, 10(10), e0140458. 26485705 10.1371/journal.pone.0140458 PMC4615632

[ece33349-bib-0040] Rottman, G. , Floyd, L. , & Viereck, R. (2004) Measurements of the solar ultraviolet irradiance In Solar variability and its effects on climate Pap J. M., Fox P., Frohlich C., Hudson H. S., Kuhn J., McCormack., North G., Sprigg W., & Wu S. T., (Eds.), (pp. 111–125). Washington, D. C. American Geophysical Union.

[ece33349-bib-0041] Roulin, A. , Almasi, B. , Rossi‐Pedruzzi, A. , Ducrest, A. L. , Wakamatsu, K. , Miksik, I. , … Jenni, L. (2008). Corticosterone mediates the condition‐dependent component of melanin‐based coloration. Animal Behaviour, 75, 1351–1358.

[ece33349-bib-0042] Saenko, S. V. , Teyssier, J. , van der Marel, D. , & Milinkovitch, M. C. (2013). Precise colocalization of interacting structural and pigmentary elements generates extensive color pattern variation in Phelsuma lizards. BMC Biology, 11, 105. 24099066 10.1186/1741-7007-11-105 PMC4021644

[ece33349-bib-0043] Saino, N. , Canova, L. , Costanzo, A. , Rubolini, D. , Roulin, A. , & Møller, A. P. (2013). Immune and stress responses covary with melanin‐based coloration in the barn swallow. Evolutionary Biology, 40, 521–531.

[ece33349-bib-0044] San‐Jose, L. M. , & Fitze, P. S. (2013). Corticosterone regulates multiple colour traits in Lacerta [Zootoca] vivipara males. Journal of Evolutionary Biology, 26, 2681–2690. 24118447 10.1111/jeb.12265

[ece33349-bib-0045] San‐Jose, L. M. , Granado‐Lorencio, F. , Sinervo, B. , & Fitze, P. S. (2013). Iridophores and not carotenoids account for chromatic variation of carotenoid‐based coloration in common lizards (*Lacerta vivipara*). American Naturalist, 181, 396–409. 10.1086/669159 23448888

[ece33349-bib-0046] Sato, T. (1968). A modified method for lead staining of thin sections. Journal of Electron Microscopy (Tokyo), 17, 158–159. 4177281

[ece33349-bib-0047] Smith, K. R. , Cadena, V. , Endler, J. A. , Kearney, M. R. , Porter, W. P. , & Stuart‐Fox, D. (2016). Color change for thermoregulation versus camouflage in free‐ranging lizards. American Naturalist, 188, 668–678. 10.1086/688765 27860512

[ece33349-bib-0048] Steffen, J. E. , & McGraw, K. J. (2009). How dewlap color reflects its carotenoid and pterin content in male and female brown anoles (*Norops sagrei*). Comparative Biochemistry and Physiology – B Biochemistry and Molecular Biology, 154, 334–340. 19647090 10.1016/j.cbpb.2009.07.009

[ece33349-bib-0049] Stevens, M. , Párraga, C. A. , Cuthill, I. C. , Partridge, J. C. , & Troscianko, T. S. (2007). Using digital photography to study animal coloration. Biological Journal of the Linnean Society, 90, 211–237.

[ece33349-bib-0050] Stuart‐Fox, D. M. , Moussalli, A. , Johnston, G. R. , & Owens, I. P. F. (2004). Evolution of color variation in dragon lizards: Quantitative tests of the role of crypsis and local adaptation. Evolution, 58, 1549–1559. 15341157 10.1111/j.0014-3820.2004.tb01735.x

[ece33349-bib-0051] Svensson, P. , & Wong, B. (2011). Carotenoid‐based signals in behavioural ecology: A review. Behaviour, 148, 131–189.

[ece33349-bib-0052] Teasdale, L. C. , Stevens, M. , & Stuart‐Fox, D. (2013). Discrete colour polymorphism in the tawny dragon lizard (*Ctenophorus decresii*) and differences in signal conspicuousness among morphs. Journal of Evolutionary Biology, 26, 1035–1046. 23495663 10.1111/jeb.12115

[ece33349-bib-0053] Teyssier, J. , Saenko, S. V. , van der Marel, D. , & Milinkovitch, M. C. (2015). Photonic crystals cause active colour change in chameleons. Nature Communications, 6, 6368. 10.1038/ncomms7368 PMC4366488 25757068

[ece33349-bib-0054] Vorobyev, M. , & Osorio, D. (1998). Receptor noise as a determinant of colour thresholds. Proceedings of the Royal Society B: Biological Sciences, 265, 351–358. 9523436 10.1098/rspb.1998.0302 PMC1688899

[ece33349-bib-0055] Vorobyev, M. , Osorio, D. , Bennett, A. T. D. , Marshall, N. J. , & Cuthill, I. C. (1998). Tetrachromacy, oil droplets and bird plumage colours. Journal of Comparative Physiology – A Sensory, Neural, and Behavioral Physiology, 183, 621–633. 10.1007/s003590050286 9839454

[ece33349-bib-0056] Vukusic, P. , Hallam, B. , & Noyes, J. (2007). Brilliant whiteness in ultrathin beetle scales. Science, 315, 348. 17234940 10.1126/science.1134666

[ece33349-bib-0057] Weiss, S. L. , Foerster, K. , & Hudon, J. (2012). Pteridine, not carotenoid, pigments underlie the female‐specific orange ornament of striped plateau lizards (*Sceloporus virgatus*). Comparative Biochemistry and Physiology – B Biochemistry and Molecular Biology, 161, 117–123. 22036614 10.1016/j.cbpb.2011.10.004

[ece33349-bib-0058] Weiss, S. L. , Mulligan, E. E. , Wilson, D. S. , & Kabelik, D. (2013). Effect of stress on female‐specific ornamentation. Journal of Experimental Biology, 216, 2641–2647. 23531828 10.1242/jeb.080937

[ece33349-bib-0059] Wingfield, J. C. , Maney, D. L. , Breuner, C. W. , Jacobs, J. D. , Lynn, S. , Ramenoffsky, M. , & Richardson, R. D. (1998). Ecological bases of hormone—behavior interactions: the “emergency life history stage”. American Zoologist, 38, 191–206.

[ece33349-bib-0060] Yewers, M. S. C. , Jessop, T. S. , & Stuart‐Fox, D. (2017). Endocrine differences among colour morphs in a lizard with alternative behavioural strategies. Hormones and Behavior, 93, 118–127. 28478216 10.1016/j.yhbeh.2017.05.001

[ece33349-bib-0061] Yewers, M. S. , McLean, C. A. , Moussalli, A. , Stuart‐Fox, D. , Bennett, A. T. D. , & Knott, B. (2015). Spectral sensitivity of cone photoreceptors and opsin expression in two colour‐divergent lineages of the lizard *Ctenophorus decresii* . Journal of Experimental Biology, 218, 2979. 26400982 10.1242/jeb.131854

[ece33349-bib-0062] Yewers, M. S. C. , Pryke, S. , & Stuart‐Fox, D. (2016). Behavioural differences across contexts may indicate morph‐specific strategies in the lizard *Ctenophorus decresii* . Animal Behaviour, 111, 329–339.

[ece33349-bib-0063] Ziegler, I. (2003). The pteridine pathway in zebrafish: Regulation and specification during the determination of neural crest cell‐fate. Pigment Cell Research, 16, 172–182. 12753383 10.1034/j.1600-0749.2003.00044.x

