# Supplementary figures and images for: Stress‐induced changes in color expression mediated by iridophores in a polymorphic lizard
Source: Ecol Evol. 2017 Sep 7;7(20):8262–72. doi: 10.1002/ece3.3349 (PMC5648675; doi:10.1002/ece3.3349)

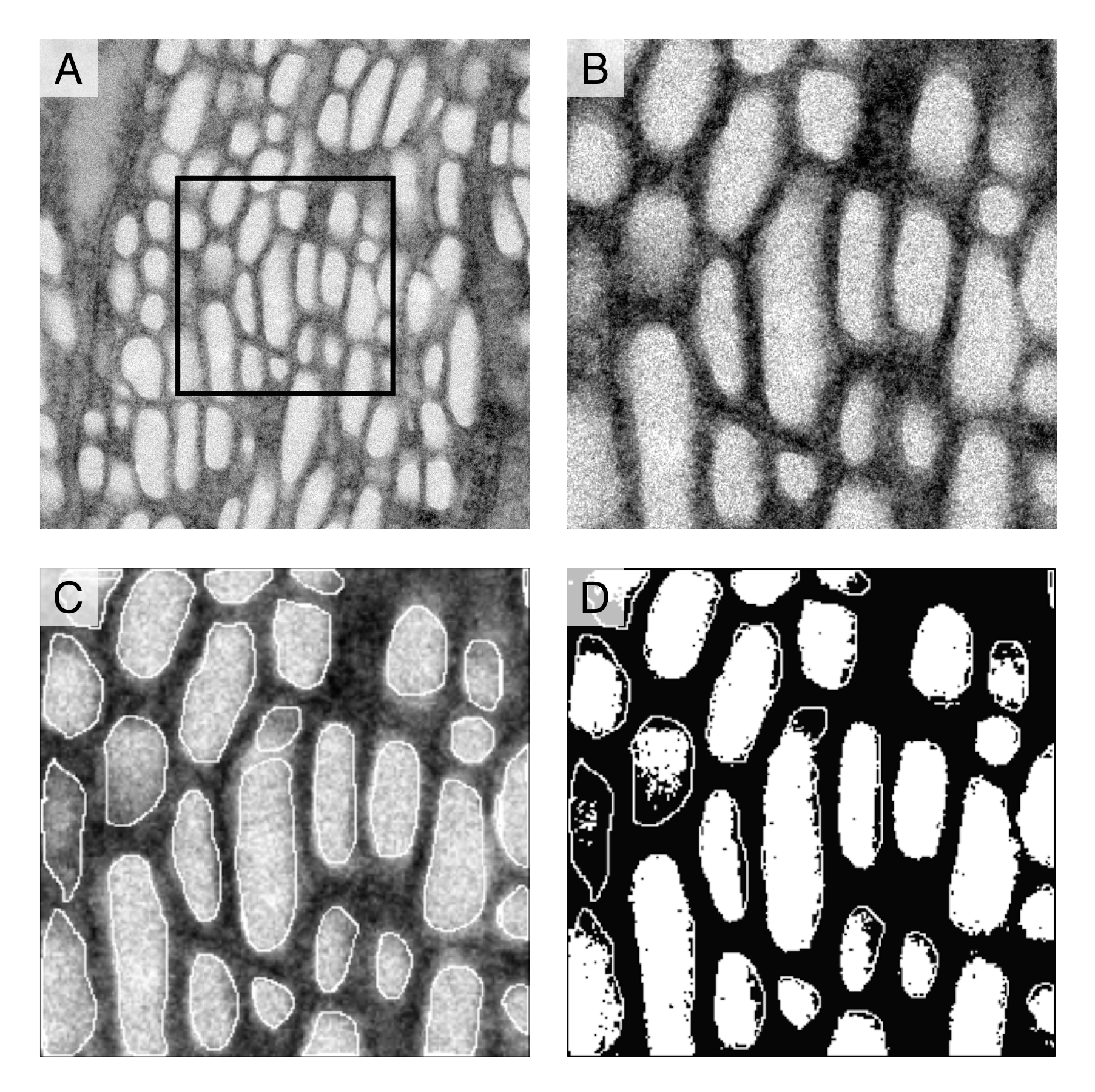

Supplement: Supplementary file 1 [file ECE3-7-8262-s001.tif]

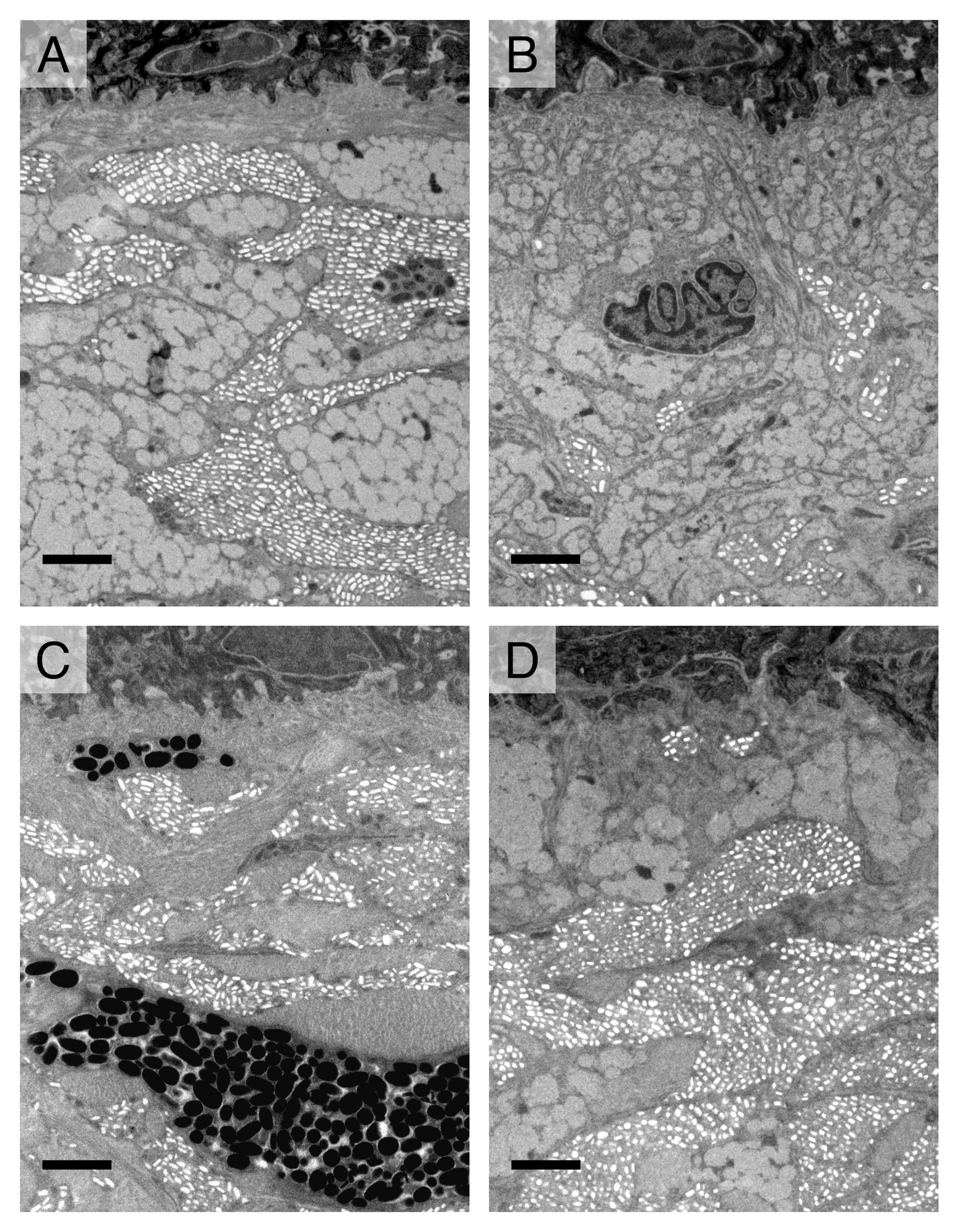

Supplement: Supplementary file 2 [file ECE3-7-8262-s002.tif]

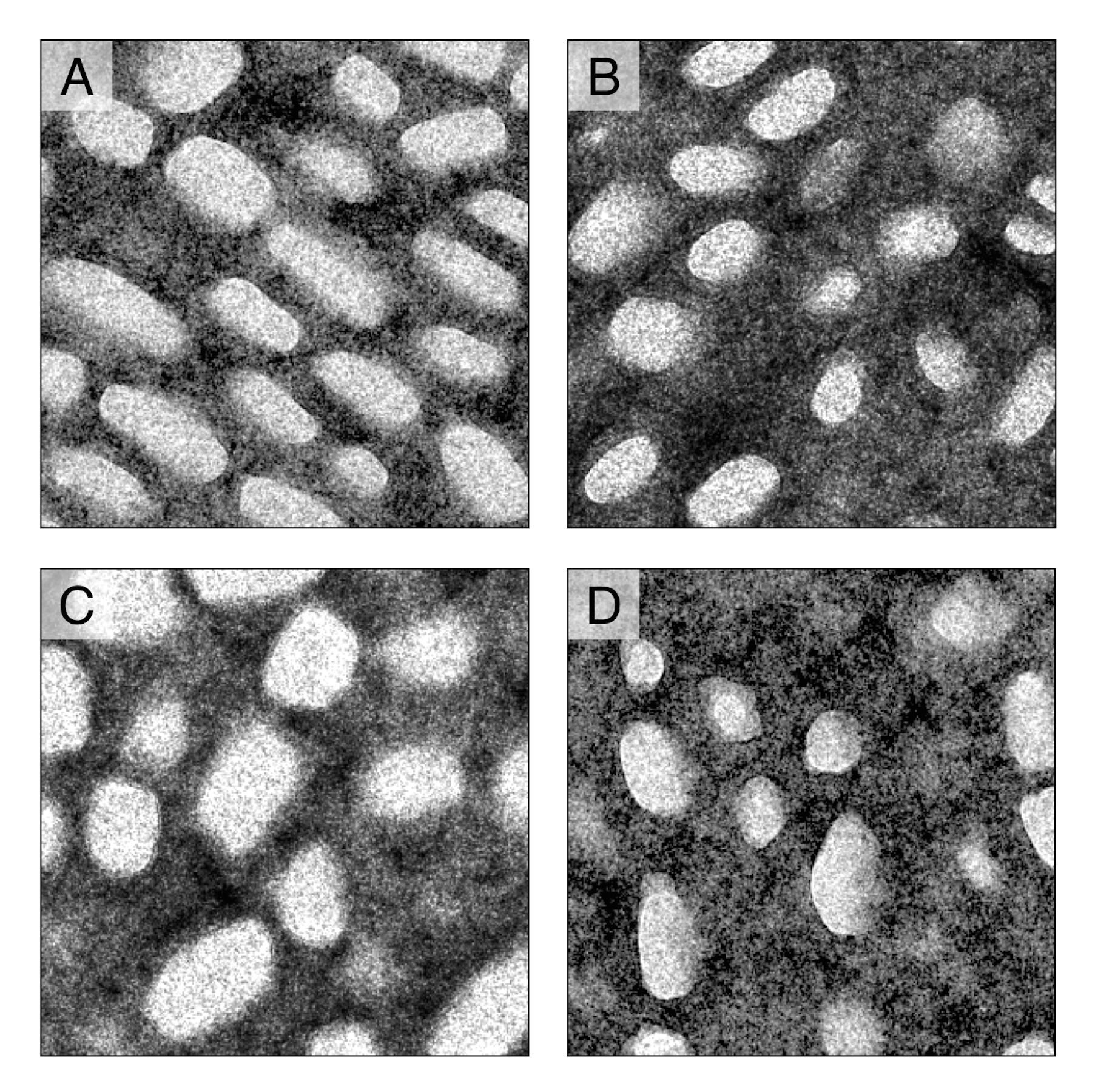

Supplement: Supplementary file 3 [file ECE3-7-8262-s003.tif]
