# Supplementary material for: Stress‐induced changes in color expression mediated by iridophores in a polymorphic lizard
Source: Ecol Evol. 2017 Sep 7;7(20):8262–72. doi: 10.1002/ece3.3349 (PMC5648675; doi:10.1002/ece3.3349)
Supplement: Supplementary file 4 [file ECE3-7-8262-s004.docx]

**Supplementary Tables and Figures**

**Table S1.** Effect of corticosterone implantation on components of throat colouration for the subset of samples selected for transmission electron microscopy.

| Dependent | Fixed factor | F_df_ | p-value |
| --- | --- | --- | --- |
| Chromatic contrast | Treatment | 0.01_1,62_ | 0.92 |
|  | Time | 1.72_2,62_ | 0.19 |
|  | Colour | 22.28_3,62_ | < 0.0001 |
|  | Treatment x Time | 0.11_2,62_ | 0.89 |
|  | Treatment x Colour | 0.66_3,62_ | 0.58 |
| Achromatic contrast | Treatment | 0.01_1,62_ | 0.94 |
|  | Time | 2.81_2,62_ | 0.07 |
|  | Colour | 23.14_3,62_ | < 0.0001 |
|  | Treatment x Time | 0.76_2,62_ | 0.47 |
|  | Treatment x Colour | 2.32_3,62_ | 0.08 |
|  |  |  |  |

**Figure S1.** The process of measuring the area of iridophore platelets within 1 x 1 µm. **A** the original image with square indicating the 1 x 1 µm plot. **B** 1 x 1 µm plot. **C** intact and partially intact platelets are outlined in white. **D** following threshold adjustment to allow selection of each platelet for measurement.

**Figure S2.** Examples of the dermal chromatophore units of *C. decresii* skin tissue. **A** yellow skin. **B** orange skin. **C** grey skin. **D** cream skin. Bar = 2 µm.

**Figure S3.** Platelets within 1 x 1 µm plots in the iridophore cells of *C. decresii* skin tissue. **A** yellow skin. **B** orange skin. **C** grey skin. **D** cream skin.
